# Analysis of MMP2 promoter polymorphisms in childhood obesity

**DOI:** 10.1186/1756-0500-4-253

**Published:** 2011-07-21

**Authors:** Angharad R Morgan, Dug Yeo Han, John MD Thompson, Edwin A Mitchell, Lynnette R Ferguson

**Affiliations:** 1 Discipline of Nutrition, FMHS, The University of Auckland, New Zealand; 2 Nutrigenomics New Zealand, New Zealand; 3 Department of Paediatrics, FMHS, The University of Auckland, New Zealand

**Keywords:** childhood obesity, percentage body fat, matrix metalloproteinase-2, genetic association

## Abstract

**Background:**

Several lines of evidence suggest a possible functional role of Matrix metalloproteinase -2 (MMP-2) in obesity. The aim of this study was to evaluate the role of MMP-2 promoter polymorphisms in percentage body fat (PBF) as a measure of childhood obesity in a New Zealand population.

**Findings:**

546 samples from the Auckland Birthweight Collaborative (ABC) study were genotyped for the three MMP-2 promoter SNPs -1306 C/T (rs243865), -1575G/A (rs243866) and -790 T/G (rs243864) using the Sequenom genotyping platform. The results demonstrated that an MMP-2 promoter haplotype is associated with PBF in New Zealand 7 year old children.

**Conclusion:**

We have previously determined that environmental factors are associated with differences in PBF in this study group, and now we have demonstrated a possible genetic contribution.

## Introduction

Obesity is reaching epidemic proportions worldwide and is now occurring at younger ages. In New Zealand, one in twelve youngsters aged 2 to 14 years are considered to be obese and one in five are classified as overweight (A Portrait of Health, 2006/07 New Zealand Health Survey).

Obese children are at increased risk of a number of health problems including diabetes, sleep problems, joint problems, early puberty or menarche, asthma and other respiratory problems [1]. Obesity often persists into adulthood and is associated with increased morbidity and mortality [2,3].

Body mass index (BMI) is the most common method used to determine obesity. However, although BMI may facilitate an adult obesity diagnosis, using this method to diagnose obesity in children may yield more ambiguous results, as BMI is a marker of relative weight and doesn't directly measure body fat [4]. Measuring percentage body fat (PBF) may be a more effective method of diagnosing obesity in children.

Childhood obesity is thought to be the result of an interplay between many genetic and environmental factors. We have previously investigated environmental factors in the Auckland Birthweight Collaborative (ABC) study, and found maternal overweight/obesity, maternal age, female gender, sedentary activity time and hours of television viewing to be independently associated with PBF in 7 year old children [5]. We are now interested in identifying possible genetic factors.

Several lines of evidence suggest a possible functional role of Matrix metalloproteinase -2 (MMP-2) in obesity. The main role of MMP-2 is in the degradation of type IV collagen, the major structural component of basement membranes. However, the enzyme also has activity toward a spectrum of functional molecules including growth factor-binding proteins and growth factor receptors, which are known to be involved in obesity. For example, MMP-2 can cleave insulin-like growth factor-binding proteins and release insulin-like growth factors [6]. It has also been suggested that MMP-2 plays an important role in adipose tissue development [7-10]. Furthermore, tissue degradation by MMP-2 is pivotal to inflammation [11], and obesity is associated with low grade inflammation.

We undertook an association analysis of 3 functional polymorphisms in the promoter of the MMP-2 gene (-1306C/T, -1575G/A and -790T/G), to investigate the possible role for MMP-2 as a genetic risk factor for obesity (in terms of increased PBF) in the ABC study.

The -1306 C/T (rs243865) polymorphism is located in the CCACC box of the Sp1-binding site and displays a strikingly lower promoter activity with the T allele [12]. The -1575G/A (rs243866) variant is located immediately 5' to a half-palindromic potential oestrogen receptor binding site and the G allele functions as an enhancer [13]. The -790 T/G (rs243864) SNP is located in the inverted GATA-1 element (CTATCT) in the promoter region. Three important transcription factors (GKLF-gut enriched Krueppel-like factor, S8, and Evil-ectopic viral integration site 1 encoded factor) may bind with the T allele but not with the G allele [14].

## Methods

### Participants

The ABC study was designed as a case-control study to determine risk factors for small for gestational age (SGA) infants and has been described in detail previously [15]. Data have been collected at birth, 1, 3.5, 7 and most recently 11 years of age. The original sample at birth resulted in a sample of 1714 subjects, of which 871 mothers were identified in the obstetric data to be of European ethnicity. At the age of 1 and 3.5 follow up of non-European ethnicities was poor resulting in a lack of ability to generalise the results from these children to their particular populations. As a result follow-up from the age of 7 has only been carried out on those children whose mothers were identified as European ethnicity at birth.

Percentage body fat (PBF) at age 7 years was the outcome measure analysed in this paper. This was measured using bioelectrical impedance analysis (BIA). This allows calculation of fat free mass which was calculated according to the formula of Schaeffer et al [16] and consequently percentage body fat.

At 11 years 546 participants consented to collection of peripheral blood (n = 397) or a buccal swab (n = 149) for DNA extraction and genotyping.

### Genotyping

DNA was extracted from the blood/buccal samples using Qiagen's DNA extraction kit and following the manufacturer's instructions. Genotyping was performed with the MassARRAY and iPlex systems of the Sequenom genotyping platform (Sequenom, San Diego, CA), which uses the MALDI-TOF primer extension assay [17,18], according to manufacturers' recommendations. The SNPs were in a multiplex with SNPs from a different study that used the same samples. Assays were optimized in 24 samples consisting of 20 reference Centre d'Etude du Polymorphisme Humain (CEPH) samples and 4 blanks. All sample plates contained cases, controls, blanks, CEPH and duplicate samples. Quality control measures included independent double genotyping, blind to sample identity and blind to the other caller, and comparison of our CEPH genotypes to those in the HapMap http://www.hapmap.org.

### Statistical analysis

SNPs were tested for deviation from Hardy-Weinberg equilibrium (HWE) using a chi-square goodness-of-fit test.

A Generalised linear model (GLM) was used to test the linearity of genotype-phenotype relationship for quantitative traits. For linearity of the genotype-phenotype relationship for quantitative traits, each SNP was coded 0, 1, and 2 for each tested allele [19,20]. For example, G/G, G/T, and T/T were coded as 0, 1, and 2 for the T allele for rs243864. All analyses were controlled for SGA status due to the disproportionate sample of SGA and AGA at birth (phase 1). Multivariable analyses also controlled for environmental factors previously shown to be independently related in this dataset to PBF namely sex of the child, maternal body mass index, maternal age at the birth of the child, hours of television watched per day, and the amount of time spent in sedentary activities [5].

To determine LD (linkage disequilibrium) between the SNPs we uploaded our data into haploview [21]. Haplotype blocks were defined using the default algorithm which uses confidence intervals [22]. Haplotype analysis was carried out using HAPLO.SCORE in R to test for association of the haplotypes with PBF.

Statistical analyses were carried out using R [23] and SAS (V9.1 SAS Institute., Cary, NC, USA). A p-value less than 0.05 was considered statistically significant.

### Ethical approval

The study received ethical approval from the Northern Regional Ethics Committee. Signed consent for the study and extraction of DNA was given by the parents of the children and assent also given by the child.

## Results

The genotype counts (frequencies) for the three genotyped SNPs are shown in table 1. Each of the SNPs met Hardy Weinberg criteria. All 3 SNPs were found to be associated with PBF (table 2). The T allele of rs243864, the C allele of rs243865 and the G allele of rs243866 were associated with a higher PBF as compared to the alleles G, T and A respectively. However, the results just fell below statistical significance after adjustment for environmental factors previously shown to be independently related in this dataset to PBF: sex of the child, maternal body mass index, maternal age at the birth of the child, hours of television watched per day, and the amount of time spent in sedentary activities [5] (table 3). The SNPs were not associated with any of the confounding factors (data not shown, but available on request).

**Table 1:** Genotype counts (frequencies) for the MMP2 promoter SNPs in the ABC samples

rs243866:	AA = 31 (0.060)	AG = 191 (0.350)	GG = 305 (0.590)
rs243865:	TT = 34 (0.066)	CT = 179 (0.347)	CC = 303 (0.587)
rs243864:	GG = 33 (0.063)	GT = 185 (0.350)	TT = 310 (0.587)

**Table 2:** Effect of MMP2 promoter SNPs on PBF

SNP	Tested allele	Estimate (95% CI)	p
rs243864	T	1.32 (0.08 - 2.56)	**0.037**
rs243865	C	1.40 (0.16 - 2.64)	**0.027**
rs243866	G	1.42 (0.16 - 2.69)	**0.028**

**Table 3:** Effect of MMP2 promoter SNPs on PBF after adjustment for confounding factors

SNP	Tested allele	Estimate (95% CI)	p
rs243864	T	0.990 (-0.19 - 2.17)	0.099
rs243865	C	1.144 (-0.03 - 2.31)	0.055
rs243866	G	1.164 (-0.03 - 2.36)	0.057

**Figure 1:**
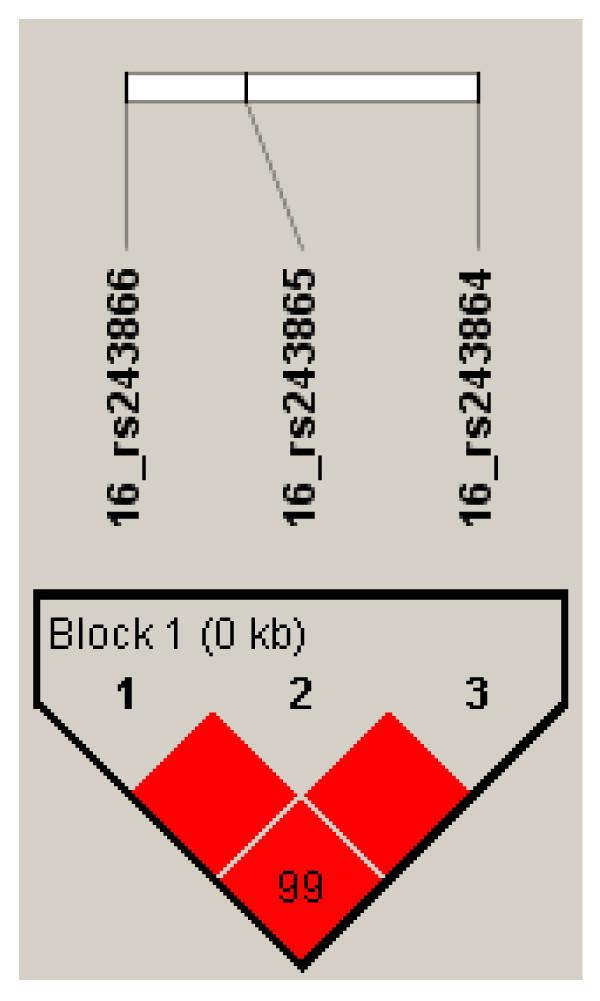
**LD plot for MMP-2 promoter SNPs**. LD (D') plot of *MMP2 *genotyped SNPs. LD between rs243866 and rs243865: D' = 1, r^2^ = 1. LD between rs243865 and rs243864: D' = 1, r^2^ = 0.989. LD between rs243866 and rs243864: D' = 0.994, r^2^ = 0.983.

The 3 MMP-2 promoter SNPs are in high LD with each other (Figure 1), so we undertook haplotype analysis. The GTA (rs243864, rs243865, and 243866 respectively) haplotype was significantly associated with PBF (p = 0.019). Individuals with the GTA haplotype had lower PBF in comparison to individuals with the TCG haplotype (table 4). After adjustment for confounding factors, the GTA haplotype remained significantly associated with PBF (p = 0.040) (table 5).

**Table 4:** Haplotype analysis

	rs243864	rs243865	rs243866	hap.freq	coef	se	p
haplo.base	T	C	G	0.766	23.63	0.71	
mmp2hap.1	G	T	A	0.231	-1.56	0.66	**0.019**
mmp2hap.rare	*	*	*	0.003	2.11	5.62	0.708
centileNT (AGA)					2.65	0.82	0.001

**Table 5:** Haplotype analysis after adjustment for confounding factors

	rs243864	rs243865	rs243866	hap.freq	coef	se	p
haplo.base	T	C	G	0.766			
mmp2hap.1	G	T	A	0.232	-1.30	0.63	**0.040**
mmp2hap.rare	*	*	*	0.003	3.17	5.33	0.553

## Discussion

This paper reports for the first time the role of MMP-2 promoter polymorphisms in childhood obesity as measured using PBF. Although we were unable to demonstrate an association for the 3 SNPs individually after correcting for confounding factors, the GTA (rs243864, rs243865, 243866) haplotype was statistically significant. It has previously been suggested that studying haplotypes could be more informative than the study of individual SNPs [24,25].

Genetic variation in MMP-2 has been previously investigated for association with obesity, in adults from Korea [26]. Whereas we have focussed on promoter SNPs and PBF, Han et al. investigated coding SNPs and looked for association with BMI. They found 2 SNPs (out of the 5 investigated) to be associated with overweight/obesity, and also identified a haplotype as significant. Taken together with our own results, it can be concluded that MMP-2 may be an important susceptibility gene for obesity in both children and adults.

The genetic association we report in the MMP-2 promoter may result from altered MMP expression, as the 3 SNPs investigated are all functional SNPs capable of affecting transcriptional activity. Indeed, associations with MMP-2 circulating levels in mouse models and obese children and adults have been previously reported. High expression of MMP-2 has been demonstrated in adipose tissue of mice with nutritionally induced obesity, as well as in genetically obese mice [9,10,27]. Bouloumiè et al. [8] provided the first evidence that human adipose tissue releases MMP-2. Later two studies by the same group described decreased circulating levels of MMP-2 (and increased levels of MMP-9 and TIMP-1) in obese children [28,29]. Whilst a study examining obesity in adults demonstrated increased MMP-2 (and MMP-9) levels [30].

Additional studies are now required to further investigate MMP-2 involvement in the development of obesity. It may be also be worth considering other matrix metalloproteinases.

In conclusion the results presented here demonstrate that an MMP-2 promoter haplotype is associated with PBF in New Zealand 7 year old children. We have previously determined that environmental factors are associated with differences in PBF in the ABC study, and now we have demonstrated a possible genetic contribution.

## Competing interests

The authors declare that they have no competing interests.

## Authors' contributions

ARM made substantial contributions to this study. She designed the study, carried out the genotyping experiments and was primary author of the manuscript. DYH was responsible for the statistical analysis. JMDT helped in establishing the ABC cohort and its data collection and contributed to editing of the manuscript. EAM established the ABC cohort and its data collection and contributed to editing of the manuscript. LRF participated in editing of the manuscript. All authors read and approved the final manuscript.
